# Immunohistochemical Analysis of Scarring Trachoma Indicates Infiltration by Natural Killer and Undefined CD45 Negative Cells

**DOI:** 10.1371/journal.pntd.0004734

**Published:** 2016-05-24

**Authors:** Victor H. Hu, Philip J. Luthert, Tamsyn Derrick, James Pullin, Helen A. Weiss, Patrick Massae, Tara Mtuy, William Makupa, David Essex, David C. W. Mabey, Robin L. Bailey, Martin J. Holland, Matthew J. Burton

**Affiliations:** 1 International Centre for Eye Health, Department of Clinical Research, Faculty of Infectious and Tropical Diseases, London School of Hygiene and Tropical Medicine, London, United Kingdom; 2 Kilimanjaro Christian Medical Centre, Moshi, Tanzania; 3 UCL Institute of Ophthalmology, London, United Kingdom; 4 Department of Clinical Research, Faculty of Infectious and Tropical Diseases, London School of Hygiene and Tropical Medicine, London, United Kingdom; 5 Department of Infectious Disease Epidemiology, Faculty of Epidemiology and Population Health, London School of Hygiene and Tropical Medicine, London, United Kingdom; University of California San Diego School of Medicine, UNITED STATES

## Abstract

**Introduction:**

The phenotype and function of immune cells infiltrating the conjunctiva in scarring trachoma have yet to be fully characterized. We assessed tissue morphology and immunophenotype of cellular infiltrates found in trachomatous scarring compared to control participants.

**Methodology:**

Clinical assessments and conjunctival biopsy samples were obtained from 34 individuals with trachomatous scarring undergoing trichiasis surgery and 33 control subjects undergoing cataract or retinal detachment surgery. Biopsy samples were fixed in buffered formalin and embedded in paraffin wax. Hematoxylin and eosin (H&E) staining was performed for assessment of the inflammatory cell infiltrate. Immunohistochemical staining of single markers on individual sections was performed to identify cells expressing CD3 (T-cells), CD4 (helper T-cells), CD8 (suppressor/cytotoxic T-cells and Natural Killer, NK, cells), NCR1 (NK cells), CD20 (B-cells), CD45 (nucleated hematopoietic cells), CD56 (NK and T-cells), CD68 (macrophages/monocytes) and CD83 (mature dendritic cells). The degree of scarring was assessed histologically using cross-polarized light to visualize collagen fibres.

**Principle Findings:**

Scarring, regardless of clinical inflammation, was associated with increased inflammatory cell infiltrates on H&E and CD45 staining. Scarring was also associated with increased CD8+ and CD56+ cells, but not CD3+ cells, suggestive of a NK cell infiltrate. This was supported by the presence of NCR1+ cells. There was some increase in CD20+ cells, but no evidence for increased CD4+, CD68+ or CD83+ cells. Numerous CD45 negative cells were also seen in the population of infiltrating inflammatory cells in scarred conjunctiva. Disorganization of the normal collagen architecture was strongly associated with clinical scarring.

**Conclusions/Significance:**

These data point to the infiltration of immune cells with a phenotype suggestive of NK cells in conjunctival trachomatous scarring. A large proportion of CD45 negative inflammatory cells were also present. Future work should seek to understand the stimuli leading to the recruitment of these cells and their role in progressive scarring.

## Author Summary

Trachoma is initiated by repeated infection of the conjunctiva throughout childhood by the bacterium *Chlamydia trachomatis* (Ct). Conjunctival inflammation and scarring progress throughout the lives of many adults even in the absence of Ct infection, causing the eyelashes to turn inwards (trichiasis) and damage the cornea, resulting in severe pain and eventually leading to blindness. The factors sustaining the inflammation that drives scarring are not understood and there is no treatment to halt scarring progression. We sought to define the phenotypes of immune cells infiltrating the conjunctiva during trichiasis. Eyelid tissue from 34 individuals with trichiasis and 33 control individuals was stained with dyes or specific antibodies to distinguish immune cell subsets. Increased inflammatory cells were detected in individuals with trichiasis even when clinical signs of inflammation were not apparent. Staining of immune cell types pointed to an increased infiltration of natural killer cells in tissue from individuals with trichiasis. These cells may cause tissue damage through cytokine secretion and cell lysis. Surprisingly, a large number of infiltrating immune cells lacked the classical immune cell marker CD45. The phenotype and function of these CD45 negative cells and their role in scarring trachoma warrants further study.

## Introduction

Trachoma starts in childhood with repeated conjunctival infection by *Chlamydia trachomatis*. The infection provokes a marked inflammatory response, which can lead to cicatricial sequelae in later life: conjunctival scarring, entropion, trichiasis, corneal opacity and blindness [1]. Trachoma is still a major problem world-wide; the World Health Organization (WHO) currently estimates it is endemic in 51 countries and is responsible for the visual impairment of 2.2 million people, of whom 1.2 million are irreversibly blind [2]. There has been an encouraging reduction in the number of children with active disease over the last few decades, which is probably attributable to improved living standards and trachoma control programmes [3]. However, even in areas where the prevalence of *C*. *trachomatis* infection has been low for some time scarring complications still appear to develop and progress [4,5]. This has implications for trachoma control programmes. There may be a need for more prolonged surveillance and it is therefore important to better understand the cicatricial disease process.

The pathophysiology of the scarring sequelae of *C*. *trachomatis* infection, both in the eye and genital tract, remains unclear and various models have been proposed [6]. The “immunological” paradigm suggests that disease is the result of a cell-mediated immune process, particularly involving T-cell responses, against specific *C*. *trachomatis* antigens [7,8]. The “cellular” paradigm argues that infected epithelial cells are central in causing tissue damage through the release of pro-inflammatory cytokines, chemokines and growth factors, although this may also subsequently involve adaptive responses [9,10]. Contemporary studies have supported the role of innate immunity in the development of scarring complications and indicate the epithelium may be important in driving these innate processes [4,11,12,13,14].

A number of studies have recently suggested a role for NK cells in trachoma. NK cells represent around 10–15% of circulating lymphocytes and were historically identified as null cells or large granular lymphocytes that can lyse target cells without previous sensitisation [15,16]. They are generally considered to be part of the innate immune response as their activity is regulated through a combination of cytokines, activating and inhibitory receptors and host cell ligands which enable them to respond rapidly to danger signals such as those triggered by bacterial, viral and parasitic infections [17]. They can also have a regulatory role on both innate and adaptive immunity through the production of cytokines and pro-inflammatory mediators, notably interferon gamma (IFNγ). A microarray study of active trachoma in children found increased expression and enrichment of genes involved in NK cell activation and cytotoxicity [13]. NK cells were also found to be the major cellular source of IFNγ, a key cytokine in the immune response to *C*. *trachomatis* infection [18]. Genetic susceptibility to early conjunctival scarring is associated with polymorphism in NK cell receptors and host cell ligands [19]. Infection of epithelial cell lines with *C*. *trachomatis* render them susceptible to NK cell lysis and a murine model of *C*. *trachomatis* infection found NK cell depletion to exacerbate the course of infection [20,21,22].

Histological examination of trachomatous tissue gives a valuable opportunity to study the disease. Several previous studies have examined the histopathology of conjunctival biopsy specimens from individuals with trachoma [23,24,25,26,27,28,29,30,31,32,33]. However, these studies suffer from relatively small sample sizes and either lacked, or had very few, control samples for comparison. We have analysed conjunctival biopsies from a larger number of patients with trachomatous trichiasis and control subjects recruited from the same geographic region. We characterised in detail the morphological and cellular changes using defined grading systems with robust analytical techniques and observers masked to case-control status. We studied whether the conjunctiva in trachomatous scarring is characterised by connective tissue changes that can be seen histologically and with an increased inflammatory cell infiltrate. We also wanted to examine the nature of this cellular infiltrate.

## Methods

### Ethical permission

This study adhered to the tenets of the Declaration of Helsinki. It was approved by the Tanzanian National Institute of Medical Research Ethics Committee, the Kilimanjaro Christian Medical Centre Ethics Committee and the London School of Hygiene and Tropical Medicine Ethics Committee. The study was explained to potential study subjects and written, informed consent was obtained before enrolment.

### Subject recruitment

Thirty-four individuals with trachomatous trichiasis (TT) were recruited from those undergoing trichiasis surgery in the Kilimanjaro Region. Thirty-three comparison participants with healthy tarsal conjunctiva were recruited from patients undergoing cataract or retinal detachment surgery at Kilimanjaro Christian Medical Centre.

### Clinical assessment and swab sample collection

All subjects were examined by an ophthalmologist using ×2.5 loupes and a bright torch. The 1981 World Health Organization (FPC) trachoma grading system was used to grade conjunctival follicles and papillae, entropion, trichiasis and corneal opacity [34]. A more detailed, previously described, grading system was used for conjunctival scarring [35,36]. Conjunctivae were anaesthetised with preservative-free proxymetacaine 0.5% eye drops (Minims, Chauvin Pharmaceuticals). Upper tarsal conjunctival swabs were collected (Dacron polyester-tipped, Hardwood Products Company, Guildford, ME) for *C*. *trachomatis* detection and put into a dry tube. Samples were kept on ice packs until frozen later the same day at -80°C.

### Biopsy samples and staining

Eyelids were injected with 2% lignocaine (Vital Healthcare, India) and eyes were cleaned with 5% povidone iodine. Biopsy samples were taken using a 3mm trephine from the tarsal conjunctiva, 2mm from the lid margin, at the junction of the medial ⅔ and lateral ⅓ of the everted lid. Samples were immediately placed in 10% neutral buffered formalin and subsequently embedded in paraffin wax. Sections, 4 μm thick, were cut perpendicular to the conjunctival surface and stained with hematoxylin and eosion (H&E) and by immunohistochemistry with the antibodies shown in Table 1. Prior to immunohistochemical staining, sections were dewaxed and pressure-cooked for 4 minutes in de-ionized water with antigen retrieval solution (Vector Lab, UK). After incubation at room temperature with the primary antibody, sections were incubated with a biotinylated secondary antibody, followed by streptavadin-conjugated horseradish peroxidase (Dako, UK), and finally 3,3’-diaminobenzidine (Dako, UK) was used as the chromogen. Endogenous peroxidase was inhibited using Real Endogenous Peroxidase Block (Dako, UK). The slides were counterstained with Harris hematoxylin. All staining procedures were performed using the Dako autostainer (Dako, UK). Positive control samples were provided by human tonsil sections and omission of primary antibody controls were used throughout.

**Table 1:** Summary of primary antibody targets.

Target name	Specificity	Dilution	Source and type
CD3	T-cell receptor complex^1^	1:400	Dako. polyclonal, rabbit. Code no. A.0452.
CD4	Helper T-cells^2^	1:50	Dako. Monoclonal, mouse. Clone 4B12.
CD8	Cytotoxic/suppressor T-cells^3^	1:100	Dako. Monoclonal, mouse. Clone C8/144B.
CD20	B-cells	1:600	Dako. Monoclonal, mouse. Clone L26.
CD45	Nucleated hematopoietic cells	1:800	Dako. Monoclonal, mouse. Clones 2B11+PD7/26.
CD56	Natural killer cells^4^	1:100	Dako. Monoclonal, mouse. Clone 123C.
CD68	Macrophages/monocytes	1:100	Dako. Monoclonal, mouse. Clone KP1.
CD83	Mature dendritic cells	1:75	Serotec AbD. Monoclonal, mouse. Clone HB15e.
NCR1	Natural killer cells	1:1000	Abcam. Monoclonal, mouse. Clone n1D9.

CD = Cluster Differentiation marker

Incubation times were 30 minutes for all antibodies

^1^ Natural killer cells also sometimes express CD3 and CD8.

^2^ Low levels of CD4 can also be expressed on other cell types including thymocytes, T cell lymphoma cells, cytotoxic/suppressor T cells, Kupffer cells and dendritic cells.

^3^ 13 C-terminal amino acids of cytoplasmic domain of human CD8α. 32 kDa polypeptide corresponding to CD8α. CD8 is expressed mostly as a αβ heterodimer by a majority of thymocytes and by class l major histocompatibility complex restricted, mature, suppressor / cytotoxic T cells. A proportion of γδ T cells and NK cells express the CD8 αα homodimer.*

^4^ Neural cell adhesion molecule 1 (NCAM1) (antibody recognizes a protein consisting of at least two subunits. NCAM is a membrane glycoprotein which has multiple isoforms generated by alternative splicing from a single gene. The core polypeptide is the 140 kDa isoform which can be variably glycosylated. The predominant isoform in NK and T cells is the transmembrane-anchored 140 kDa protein.

### Microscopic examination of biopsy samples

Grading was performed by ophthalmic pathologists who were masked to the clinical status of the patients. The grading was performed on the subepithelial tissue (lamina propria and stroma unless otherwise stated) for the parameters below. Example images are shown in S1 Fig. The degree of scarring was assessed using cross polarised light for both the subepithelial connective tissue and the tarsus. Initial comparison of the examination of picrosirus red and other tinctorial stains under cross polarised light showed that the organization of collagen bundles was apparent with a variety of staining methods. For assessment purposes we used elastin/Van Gieson stained preparations as these sections contained all of the samples available. Tissue appearing healthy was graded as 0 and grades 1–3 used for progressive disorder of the normal appearance. Density of inflammatory cell infiltrate was assessed using the H&E stained slides and an ordinal scale of 0–3 where 0 = scattered cells, 1 = few cells, 2 = moderate cells and 3 = abundant/confluent cells. Immunohistochemistry was performed with the antibodies listed in Table 1. The whole of the available section was examined and an ordinal scale of 0–3 was used where 0 = no/very few cells, 1 = few cells, 2 = moderate cells and 3 = abundant/confluent cells.

### Data analysis

Data were entered into Access 2007 (Microsoft) and analysed using STATA 11.0 (StataCorp LP, TX). Fisher’s exact tests were used to determine the strength of association for the parameters graded by case-control status.

## Results

### Study participants

Demographic and clinical characteristics of the participants are shown in Table 2. All cases had tarsal conjunctival scarring with trichiasis, defined as at least one lash rubbing against the globe or evidence of recent epilation. The majority of the cases were of Maasai ethnicity, while the controls were more evenly divided between different ethnic groups. Clinically visible conjunctival inflammation was very strongly associated with the presence of scarring. Lymphoid follicles, which are the clinical hallmark of active disease in children, were not seen in any of the participants. Infection with *C*. *trachomatis* was not detected in any of the participants. Surgery was performed on the right eye of 14 (40%) of the cases and 19 (58%) of the controls.

**Table 2:** Demographic and clinical characteristics.

Parameter	Cases		Controls		p-value*
	N = 34		N = 33		
Demographic Characteristics					
Female [n (%)]	22	(64.7)	15	(45.5)	0.09
Age in years [ū (95%CI)]	69.6	(65.6–73.6)	67.1	(62.1–72.1)	0.52
Ethnicity [n (%)]					<0.001
Maasai	28	(82.3)	0	(0.0)	
Chagga	4	(11.8)	14	(42.4)	
Pare	0	(0.0)	8	(24.2)	
Other	2	(5.9)	11	(33.3)	
Any education [n (%)]	4	(12.5)	26	(86.7)	<0.001
Clinical Characteristics					
Scarring grade [n (%)]					
S0	-	-	33	(100)	
S1a	0	0	-	-	
S1b	0	0	-	-	
S1c	6	(17.7)	-	-	
S2	5	(14.7)	-	-	
S3	23	(68.6)	-	-	
Papillary inflammation grade [n (%)]					<0.001
P0	4	(11.8)	31	(93.9)	
P1	5	(14.7)	1	(3.0)	
P2	18	(52.9)	1	(3.0)	
P3	7	(20.6)	0	(0.0)	

* Using the Fisher’s exact test, and the Wilcoxon ranked sum test for age.

### Histological evidence of scarring

In healthy subepithelial tissue, short connective tissue fibres running parallel to the surface in an ordered manner were observed. In healthy tarsal tissue long connective tissue fibres passing between the meibomian glands, perpendicular to the mucosal surface, were seen. These terminated in the shorter fibres running parallel to the surface, giving rise to a “T” configuration [37]. On examination with cross polarised light all except one of the samples from control participants had a visible “T-sign” in the tarsal connective tissue, while all of the cases had some evidence of disorganisation of this appearance (Table 3).

**Table 3:** Connective tissue scarring grade using polarised light.

Scarring grade	Cases		Controls		p-value*
	n	(%)	n	(%)	
Subepithelial tissue	N = 29		N = 31		<0.001
0	3	(10.3)	26	(83.9)	
1	13	(44.8)	5	(16.1)	
2	12	(41.4)	0	(0.0)	
3	1	(3.5)	0	(0.0)	
Tarsal tissue	N = 13^†^		N = 32		<0.001
0	0	(0.0)	31		
1	5	(38.5)	0	(0.0)	
2	6	(46.1)	1	(3.1)	
3	2	(15.4)	0	(0.0)	

* Using Fishers’ exact test.

^†^ Insufficient tarsal tissue limited the number available for grading.

### Inflammatory cells

**Fig 1:**
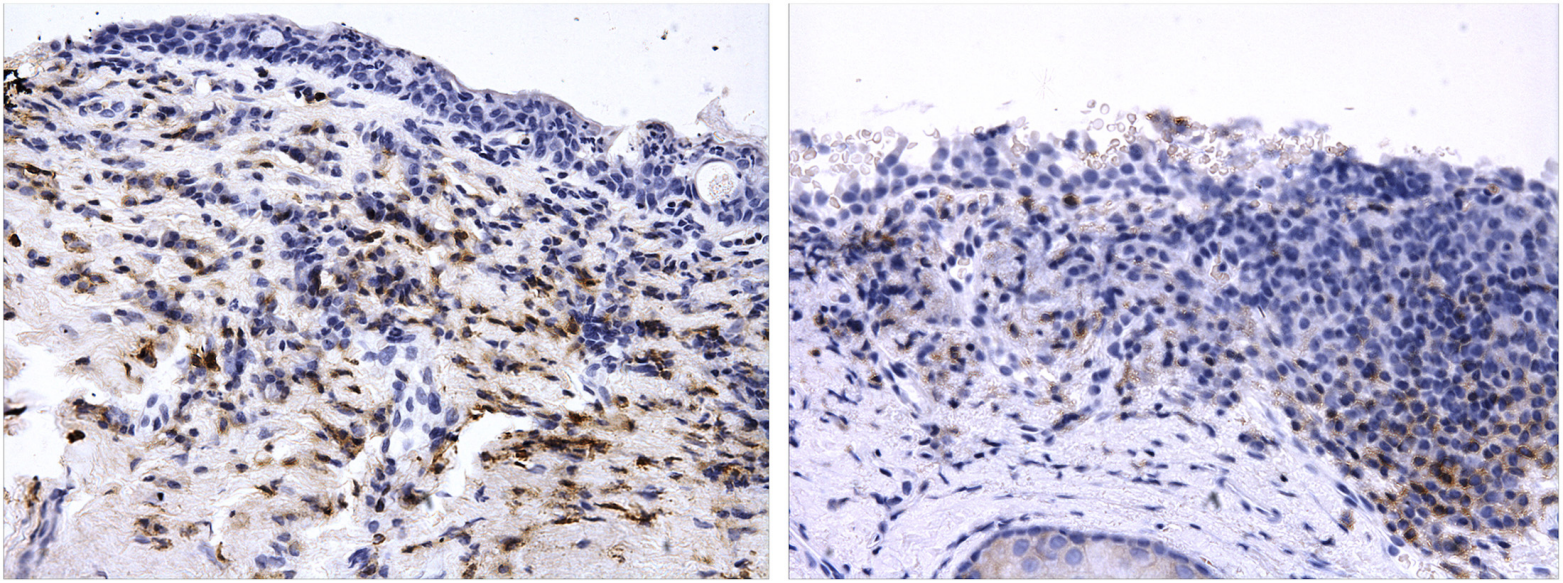
Conjunctival sections after staining for CD45. Some CD45 positive cells, stained brown, can be observed. However, many CD45 negative inflammatory cells can also be seen. Original magnification ×400.

Cases had significantly more inflammatory cells than controls, assessed both with the H&E and CD45 stains (Table 4). A number of sections were noted to have a marked inflammatory cell infiltrate in which a large proportion of the cells were CD45 negative (Fig 1). The inflammatory cell infiltrate was also analysed in relation to the presence of clinical inflammation (with significant inflammation defined as grades P2/3 and non-significant inflammation as grades P0/1). There was more infiltrate and CD45 staining in individuals with clinical inflammation (P2/3). However, if this analysis is restricted to cases only, there was no significant difference for inflammatory cell infiltrate by H&E (p = 0.59) or CD45+ cell infiltrate by IHC (p = 0.38) in inflamed *vs* non-inflamed cases (although the number of non-inflamed cases was small, n = 9). The inflammatory cell infiltrates were more marked in non-inflamed cases compared to non-inflamed controls, for both the H&E stained sections (p = 0.005) and CD45+ IHC stained sections (p = 0.001).

**Table 4:** Inflammatory cell infiltrates.

Parameter	Cases		Controls		p-value*
	n	(%)	n	(%)	
H&E inflammatory infiltrate	N = 32		N = 33		<0.001
0	0	(0.0)	16	(48.5)	
1	15	(46.9)	14	(42.4)	
2	8	(25.0)	3	(9.1)	
3	9	(28.1)	0	(0.0)	
IHC CD45+ cells	N = 34		N = 33		<0.001
0	6	(17.7)	21	(63.6)	
1	11	(32.3)	11	(33.3)	
2	9	(26.5)	0	(0.0)	
3	8	(23.5)	1	(3.0)	

* Using Fisher’s exact test.

### Characterisation of infiltrating cell immunophenotype by immunohistochemical cell staining

**Fig 2:**
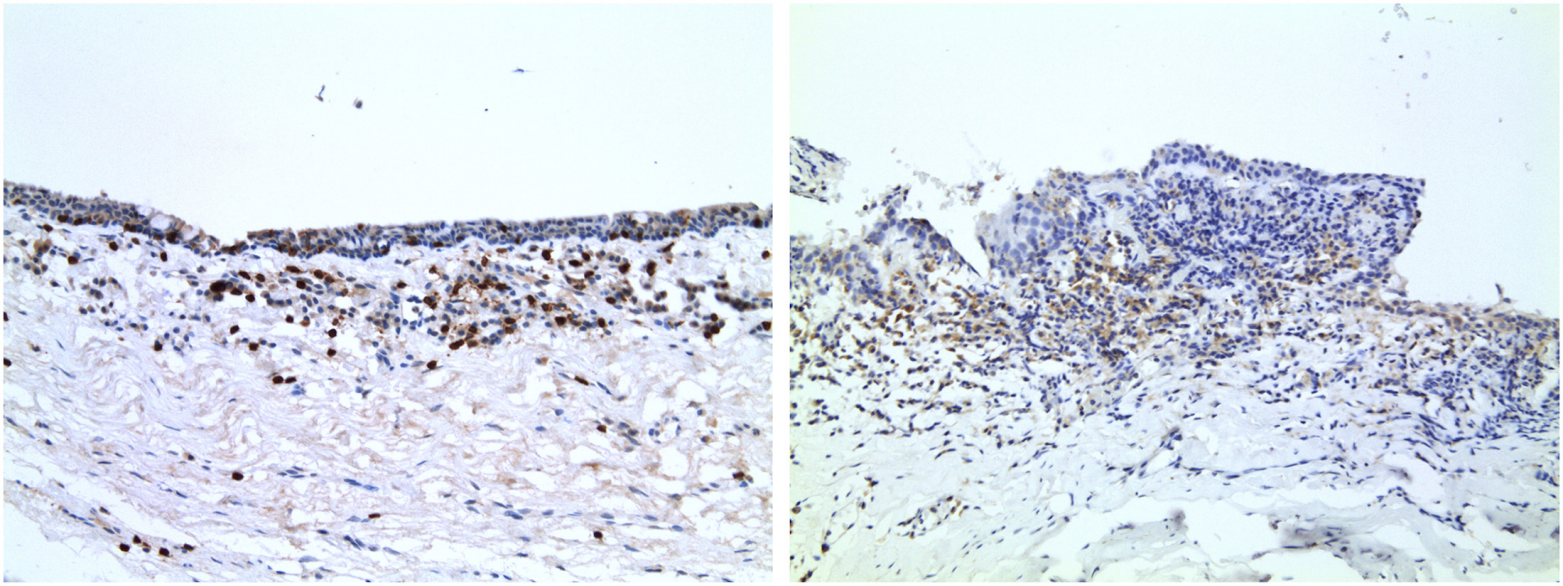
Conjunctival sections from two patients with trichiasis after staining for NCR1. Numerous NCR1 positive cells, stained brown, can be seen. Original magnification ×200.

Cases had significantly more CD8+, CD56+ and CD20+ cells than controls (Table 5). More than half of the cases had moderate levels of CD8+ cells compared to very few of the controls (p = 0.001, none of the sections were graded as 3). Almost a third of the cases had moderate levels for CD56+ cells compared to none of the controls (p = 0.001). No sections stained for CD8 or CD56 reached grade 3. Almost half of the cases had moderate or confluent levels for CD20+ cells compared to very few of the controls (p = 0.03). Of borderline statistical significance, cases had marginally more CD3+ cells than controls. There was a significant association between the presence of CD8+ and CD56+ cells (Table 6). Neutrophils (which are generally easily recognised on histological sections without specific immunohistochemical staining) were very infrequent in either cases or controls and therefore no formal grading was undertaken. Natural cytotoxicity triggering receptor 1 (NCR1) staining was undertaken after the initial analysis, when there were only a limited number of sections remaining, constraining the analysis to a more qualitative nature. Of those that were stained, however, NCR1+ cells were found more commonly in cases and in those samples where CD56+ cells were present (Fig 2). NCR1 staining using an appendix specimen as a control sample is shown in S2 Fig for comparison.

**Table 5:** Presence of immune cell markers and phenotypes by immunohistochemistry.

Cellular stain	Cases		Controls		p-value*
	n	(%)	n	(%)	
CD3	N = 33		N = 33		0.06
0	16	(48.5)	20	(60.6)	
1	12	(36.4)	13	(39.4)	
2	5	(15.1)	0	(0.0)	
3	0	(0.0)	0	(0.0)	
CD4	N = 33		N = 32		0.36
0	10	(30.3)	6	(18.8)	
1	19	(57.6)	24	(75.0)	
2	4	(12.1)	2	(6.2)	
3	0	(0.0)	0	(0.0)	
CD8	N = 33		N = 32		0.001
0	0	(0.0)	1	(3.1)	
1	14	(42.4)	27	(84.4)	
2	19	(57.6)	4	(12.5)	
3	0	(0.0)	0	(0.0)	
CD20	N = 32		N = 33		0.03
0	8	(25.0)	13	(39.4)	
1	10	(31.3)	16	(48.5)	
2	10	(31.3)	4	(12.1)	
3	4	(12.5)	0		
CD56	N = 20^†^		N = 31		0.001
0	8	(40.0)	10	(32.3)	
1	6	(30.0)	21	(67.7)	
2	6	(30.0)	0	(0.0)	
3	0	(0)	0	(0)	
CD68	N = 33		N = 33		0.33
0	23	(69.7)	23	(69.7)	
1	10	(30.3)	8	(24.2)	
2	0	(0.0)	2	(6.1)	
3	0	(0.0)	1	(0.0)	
CD83	N = 32		N = 33		0.68
0	6	(18.8)	8	(24.2)	
1	16	(50.0)	16	(48.5)	
2	10	(31.2)	8	(24.2)	
3	0	(0.0)	1	(3.0)	

* Using Fisher’s exact test.

^†^ A number of the sections were no longer available for CD56 staining owing to limited sample.

**Table 6:** Comparison of CD8 and CD56 cellular grades.

			CD56		
		0	1	2	Total
	0	1	0	0	1
CD8	1	12	20	1	33
	2	5	7	5	17
	Total	18	27	6	51

Fisher’s exact test = 0.03

## Discussion

In this study we have characterised in detail the morphological and cellular changes found in trachomatous scarring. Scarring was associated with marked infiltrates of CD8+ and CD56+ cells, compared to controls. CD56, used in conjunction with other markers such as the pan-T cell marker CD3, is used to identify NK cells, which are regarded as CD3- CD56+ [15,16,38]. NK cells lack a single defining genetic or phenotypic characteristic and share considerable overlap with cytotoxic CD8+ T-cells; the CD8 antigen is expressed by both T-cells and NK cells [38]. If the CD8+ stained cells were T-cells they would be expected to co-express CD3. However, we found a marked lack of CD3+ cells relative to CD8+ cells. This suggests that the increased CD8+ cells found in scarred conjunctiva may not be T-cells, lending weight to the possibility that they may be NK cells. This possibility is further supported by a significant association between CD8+ and CD56+ infiltrates, Table 6 (p = 0.03 with Fisher’s exact test). Additional staining for NCR1 in a more limited number of samples was also consistent with an increased presence of NK cells in scarred cases. These results overall support the hypothesis that scarred conjunctiva in trachoma contains a prominent NK cell infiltrate. To more definitively confirm this phenotype double antibody staining techniques, which present considerable technical difficulties in limited ocular samples, would be required.

While the results of the present study require confirmation, the data suggests that NK cells were increased in the conjunctivae of trichiasis cases and thus may be important in the development of scarring complications. Combinations of CD3-, CD56+, CD8+ and NCR1+ IHC staining have previously been used for the identification of NK cells in lymph nodes, colorectal cancer tissue and in the gut mucosa of HIV infected individuals [39,40,41]. Recently, a form of NK cell memory-like responses that contribute to the effector phase of vaccine-induced adaptive immune responses or display heightened responses following repeated challenge infections have been identified [42,43]. Repeated *C*. *trachomatis* infection may cause a pathological NK cell response resulting in tissue damage either through a direct cytolytic effect or through the production of cytokines and related molecules. This is consistent with recent studies pointing towards the importance of innate immune responses in the pathogenesis of trachoma and warrants further investigation into the role of NK cells [11,13,14].

Some of the findings in our study contrast with two previous immunohistological studies on biopsy samples from subjects with trachomatous scarring. Both found increased T-cell populations in scarred tissue and suggested that in inflamed cases these were predominantly CD4+ T-cells and in non-inflamed cases predominantly CD8+ T-cells [24,26]. However, these studies had a number of methodological and analytical differences which may explain the discrepancy with our conclusions. The first study recruited only three control participants whose demographic details were not presented; the grading system used was not defined; only limited data was presented and no significance testing was performed [24]. The second study appears to have been performed in a non-trachoma endemic region; it also utilised only three control samples which were collected post-mortem; cases were divided into inflamed or non-inflamed on the basis of the number of immunohistochemically stained cells present in relation to control samples (rather than clinical status); relatively limited data and no tests for significance were performed [26]. Other studies looking at cell populations in trachomatous scarring have not included control tissue or presented quantitative data [23,31].

We also found evidence that trachomatous scarring is associated with increased numbers of CD20+ B-cells. Anti-chlamydial antibodies have been found in tears and sera of both children with active disease and adults with scarring [44,45,46,47]. Whether these are protective, or simply markers of previous infection, is not known. In contrast, animal models of genital tract infection support the role of humoral immunity in protection against re-infection [48,49,50,51].

We found no evidence suggestive of increased T-helper cells (CD4+), macrophages (CD68+) or neutrophils in cases of scarring. The presence of neutrophils tends to be transient and it is possible that a significant neutrophil presence may not have been detected by our analysis. There was also no difference in the levels of cells expressing CD83+, a marker of mature dendritic cells. However, dendritic cell immunophenotyping is more complex than definitive identification of NK cells. Dendritic cell characterisation normally requires a combination of staining intensity coupled with positive and negative markers. This was beyond the scope of this study owing to the limited amount of biopsy tissue and the total number of immunohistochemical markers used.

A marked inflammatory cell infiltrate was seen in biopsies from individuals with scarring which could not be accounted for by the specific stains and increased numbers of CD8+, CD56+ and CD20+ cells. It was also notable that in a number of cases there were numerous CD45 negative inflammatory/immune cells present. Based on previous studies we expected most or all of the inflammatory cells present to be CD45+. CD45 is expressed on nearly all nucleated cells of hematopoietic origin [52]. The identity of these CD45 negative cells infiltrating scarred tissue remains an enigma. However, one of several potential explanations might be the presence of follicular dendritic cells, which are non-hematopoietic in origin and associated with lymphoid follicle formation [53,54].

Conjunctival inflammation is probably on the causal pathway to the development and progression of scarring complications in trachoma and we found the presence of scarring to be very strongly associated with clinical inflammation [4,55,56,57,58,59]. Of interest, our data showed that cases with more clinical inflammation did not have increased histological cellular infiltrates compared to cases with less clinical inflammation. These clinically non-inflamed cases did, however, have more cellular infiltrate compared to clinically non-inflamed controls. While there were small numbers involved in these comparisons, they suggest that scarring is associated with ongoing inflammatory/immune cell infiltration, even in the absence of a clinically apparent inflammatory episode characterised by hyperaemia and papillary reaction. The observation that an increased number of inflammatory cells were present in non-clinically inflamed trichiasis cases relative to controls was also observed in a parallel IHC study with a total of 36 samples, supporting this theory (Tamsyn Derrick, personal communication).

This study benefitted from a larger number of age-matched control participants than previous studies. The socio-demographic background of the controls differed to a degree from the cases, as reflected in the different educational levels and ethnicity. However, many controls were identified through outreach programmes in rural areas where trachoma is typically found and most had the cost of their surgery subsidised. Identifying unaffected control subjects with completely identical backgrounds to scarred trachoma cases is challenging as often the majority of older adults have some conjunctival scarring in endemic areas. The inclusion of control participants has allowed us to compare the normal pattern of conjunctival immune cells to that found in scarring trachoma. This comparison is essential for interpretation of diseased tissue, especially in the geographic regions where trachoma is found since these tend to have a relatively high prevalence of conjunctival non-chlamydial bacterial infection [35,60].

Earlier studies of conjunctival cell populations in healthy individuals have found that inflammatory / immune cells are normally present. T-cells are the most abundant cell type, with B-cells, macrophages, NK cells, neutrophils and dendritic cells also present [61,62,63,64]. Varying proportions of CD4+ and CD8+ cells have been reported, but with roughly overall equal numbers [61,62,63,64]. A slight predominance of CD8+ cells in the tarsal stroma was previously reported, and this is consistent with what we observed in controls.

Quantification of tarsal conjunctival scarring is not straight-forward with a paucity of robust grading systems in the literature. As part of these studies we developed a new system for grading both the sup-epithelial and tarsal connective tissue using cross-polarised light [37]. This allowed us to directly visualise collagen fibres and the grading closely corresponded to the clinical status. The association was particularly strong for grading the tarsal connective tissue where the fibres were longer and could be more easily visualised.

In conclusion, our study found evidence most consistent with a NK cell infiltrate in scarring trachoma and lends further support to studies implicating innate immune responses in the pathogenesis of trachoma. Scarred tissue was also infiltrated by large numbers of CD45 negative cells, which require further work to be characterised.

## Supporting Information

S1 Fig Example images for conjunctival histological grading. The top row shows grade 0 and the bottom row grade 3 (where available). A—CD3; B- CD4; C—CD8; D—CD20; E—CD45; F—CD56; G—CD68; H—CD83. Original magnification ×200.
(TIFF)

S2 Fig Control image from an appendix specimen. Original magnification ×200.
(TIFF)
