# Supplementary figures and images for: Immunohistochemical Analysis of Scarring Trachoma Indicates Infiltration by Natural Killer and Undefined CD45 Negative Cells
Source: PLoS Negl Trop Dis. 2016 May 24;10(5):e0004734. doi: 10.1371/journal.pntd.0004734 (PMC4878762; doi:10.1371/journal.pntd.0004734)

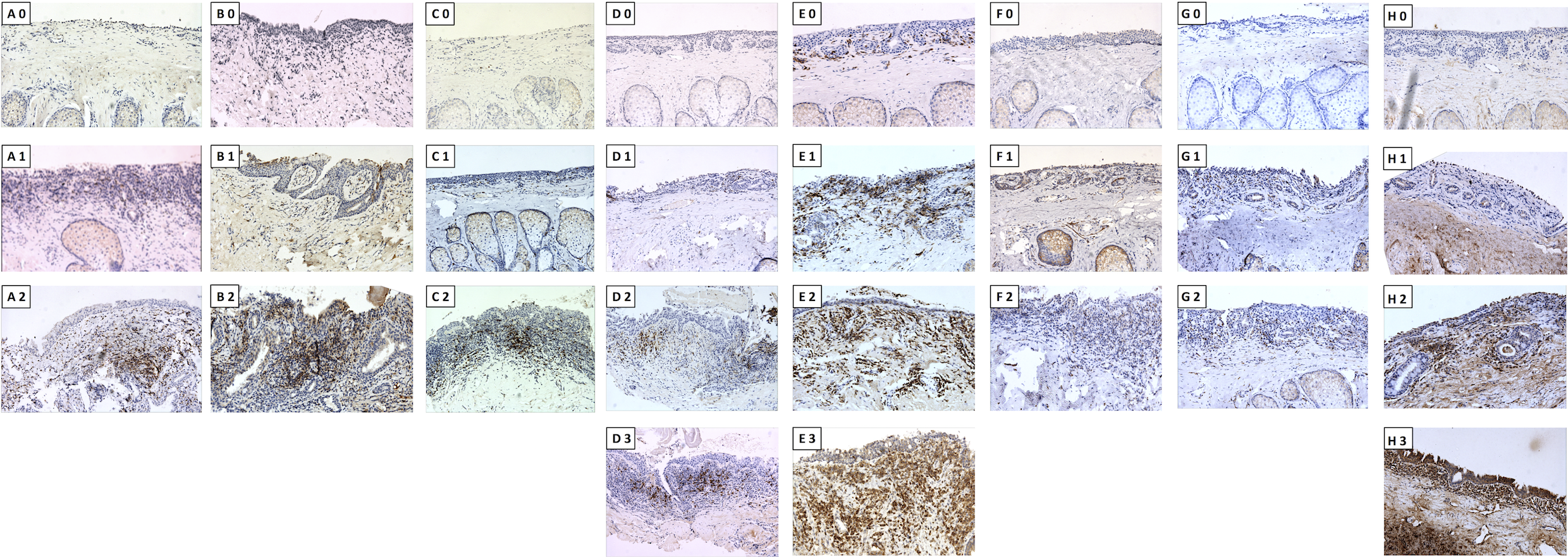

Supplement: S1 Fig — The top row shows grade 0 and the bottom row grade 3 (where available). A—CD3; B- CD4; C—CD8; D—CD20; E—CD45; F—CD56; G—CD68; H—CD83. Original magnification ×200. (TIFF) [file pntd.0004734.s001.tiff]

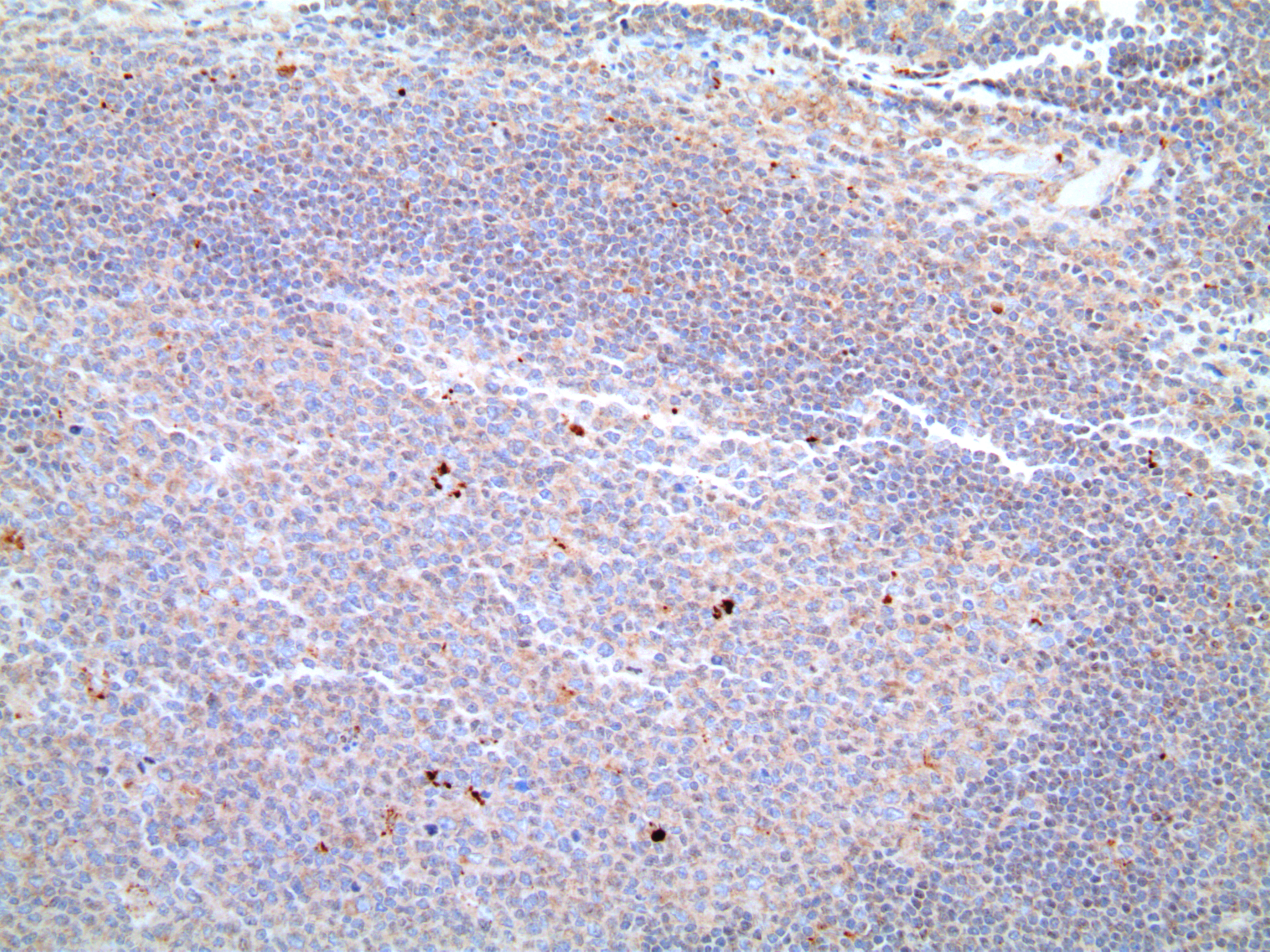

Supplement: S2 Fig — Original magnification ×200. (TIFF) [file pntd.0004734.s002.tiff]
